# piRNA DQ690018 increased bone mass by promoting the proliferation and osteogenic differentiation of BMSCs

**DOI:** 10.1186/s13018-025-06360-4

**Published:** 2025-12-07

**Authors:** Yang Li, Hui Ding, Xinyuan Liao, Xuyi Wang, De Li, Chenglong Wang

**Affiliations:** 1 Department of Bone and Joint Surgery, Department of Orthopedics, Renji Hospital, Shanghai Jiaotong University School of Medicine, Shanghai, China; 2 Present Address: Department of Orthopedic Surgery, Xinhua Hospital Affiliated to Shanghai Jiao Tong University School of Medicine (SJTUSM), Shanghai, China; 3 Present Address: Department of Gynecology of Shanghai First Maternity & Infant Hospital, Tongji University School of Medicine, Shanghai, China; 4 Department of Orthopaedics, Spine Center, Shanghai Changzheng Hospital, Second Affiliated Hospital of Naval Medical University, Shanghai, 200003 China; 5 Bengbu Medical University, Bengbu, China

**Keywords:** Bone mesenchymal stem cells, PIWI-interacting RNAs, Proliferation, Migration, Osteogenic differentiation

## Abstract

**Background:**

The proliferation, migration, and osteogenic differentiation of bone mesenchymal stem cells (BMSCs) play vital roles in maintaining bone mass and metabolism. BMSC base cell therapy has become a competitive treatment option for osteoporosis. PIWI-interacting RNAs (piRNAs) are critical regulators of cellular processes.

**Methods:**

Small RNA sequencing and qPCR were used to detect piRNA expression during BMSC proliferation. CCK-8, clone formation, and EdU assays were used to detect the effect of piRNA DQ690018 on BMSC proliferation. Wound healing and Transwell assays were used to assess the effect of piRNA DQ690018 on the migration of BMSCs. RNA sequencing was performed to identify DQ690018. Alkaline phosphatase and alizarin red staining were used to evaluate the effect of DQ690018 on osteogenic differentiation.The glucocorticoid-induced osteoporosis animal model was used to assess the impact of DQ690018 on bone mass. Micro-CT and hematoxylin and eosin staining of tibia were used to measure bone mass.

**Results:**

Fifteen piRNAs were upregulated, and 81 piRNAs were downregulated during BMSC proliferation. DQ690018 promotes BMSC proliferation by downregulating p21 expression and BMSC migration by upregulating vinculin (Vcl) expression. Furthermore, it improved the osteogenic differentiation of BMSCs and promoted the effect of BMSC base cell therapy in maintaining bone mass.

**Conclusion:**

DQ690018 is a novel target for increasing the proliferation, migration, and osteogenic differentiation of BMSCs.

**Supplementary Information:**

The online version contains supplementary material available at 10.1186/s13018-025-06360-4.

## Introduction

Stem cell-based therapy is an important treatment method in the field of regenerative medicine that may completely change the current treatment status of some diseases, significantly reduce the economic burden on patients, and improve their quality of life [1, 2]. However, due to many factors, the transplanted cells have poor viability, which limits the clinical application of cell therapy [3]. BMSCs have several advantages for tissue engineering. BMSCs are easy to obtain and source. They have strong self-replication abilities and are easy to expand. A large number of BMSCs can be obtained through short-term in vitro cultures. They exhibit low immunogenicity and good tissue compatibility. After being implanted in vivo, they adapt well to the internal environment and maintain good biological characteristics. BMSC transplantation is a very promising treatment option for large bone defects and osteoporosis. BMSCs are affected by factors such as in vitro culture, hypoxia, inflammation, and mechanical pressure. Thus, their proliferation, migration, and osteogenic differentiation are impaired, affecting the therapeutic effect of BMSCs in vivo [4, 5].

Enhancing the function of BMSCs is the key to optimizing bone injury treatment using stem cells [6]. First, it is necessary to significantly improve the expansion ability of BMSCs in vitro and obtain enough to ensure the required amount for clinical transplantation [7–9]. Second, it is necessary to ensure that the transplanted cells continue to survive and migrate to the site of injury. Finally, BMSCs must be able to differentiate into osteoblasts and release cytokines to improve the microenvironment at the injury site. At present, there are many strategies to improve the function of transplanted BMSCs, including the use of drugs such as rapamycin for treatment and the addition of cytokines such as TGF-β1 or BMP-2 [10]. These methods can significantly enhance the proliferation, migration, and osteogenic differentiation of BMSCs [11, 12]. Genetic modification is another important method for significantly enhancing the function of BMSCs [13]. Overexpression of BMP-2 and CXCR4 in BMSCs using lentiviruses can significantly enhance the osteogenic differentiation ability of BMSCs and promote the effects of BMSC therapy [14–17].

PIRNAs (PIWI-interacting RNAs) are small RNA molecules that interact with PIWI proteins. The combination of the PIWI protein with piRNA can inhibit the function of transposons through the ping-pong effect and play a role in stabilizing the genome [18]. In addition, piRNAs participate in gene regulation through histone modification and DNA methylation. PiRNA-823 maintains myeloma stem cell stemness by activating DNMT3B [19]. piRNA expression has been found in exosomes from human bone marrow mesenchymal stem cells [20]. piRNAs play an important role in the regulation of stem cell function[21].

In this study, we hypothesize that piRNAs, particularly piRNA DQ690018, play a crucial regulatory role in BMSC functions, including proliferation, migration, and osteogenic differentiation. To test this hypothesis, our objectives are as follows: (i) to establish a BMSC proliferation model by comparing the serum-free test (ST) and general medium (GM) conditions; (ii) to perform small RNA sequencing to identify piRNAs associated with BMSC proliferation; (iii) to evaluate the effect of piRNA DQ690018 on BMSC proliferation, migration, and osteogenic differentiation; and (iv) to determine the target gene(s) regulated by DQ690018. This study aims to not only elucidate the regulatory mechanism of DQ690018 on BMSC functions but also to provide a novel target for BMSC-based cell therapy..

## Methods

### BMSC proliferation

This study was approved by the Animal Ethics Committee of Xinhua Hospital Affiliated to Shanghai Jiao Tong University School of Medicine, and BMSCs were isolated from the tibial tissue of 8-week-old male C57BL/6 J mice. BMSCs were inoculated into a 6-well cell culture plate and incubated with DMEM low-glucose medium without serum for 24 h. The serum-free test (ST) group cells were cultured in a serum-free medium for 24 h, and the general medium (GM) group cells were cultured in a medium containing 10% fetal bovine serum for 24 h. Afterward, the total RNA of the BMSCs was extracted using TRIzol reagent (Biosharp #BS259A). basic-fibroblastic growth factor (bFGF; PeproTech #450–33) and insulin-like growth factor-1 (IGF-1; PeproTech #250–19) were used to stimulate the growth of BMSCs at 20 ng/mL and 100 ng/mL, respectively, for 48 h.

### RNA sequencing

Small RNA sequencing was performed by Sangon Biotech Co. Ltd. (Shanghai, China). First, a small RNA sequencing library was constructed, and comparative analysis was done using the BLAST (2.9.0 +) software. The number of read counts was quantitatively obtained from the raw sequencing data and was normalized to CPM values using the miRDeep2 software. The R vegan package was used to calculate the distance between samples and perform principal component analysis. Heat map correlations and distances between samples were drawn using the R gplots package to display sample correlations. The DESeq2 software was used to screen differential genes with a significant p value ≤ 0.05 and multiple of difference |FoldChange|≥ 2.

mRNA sequencing was performed by Hangzhou Lianchuan Biotechnology Co., Ltd. First, an mRNA library was constructed and sequenced using an Illumina Novaseq™ 6000 apparatus (LC Bio Technology Co., Ltd. Hangzhou, China) according to standard operations, with the sequencing mode set as PE150. We obtained and filtered high-quality offline sequenced data (Clean Data) and compared them to the reference genome of the project species. We then quantified each gene or transcript using StringTie.

### BMSC proliferation analysis

For CCK-8 detection, BMSCs (2000 cells/well) were first inoculated and allowed to adhere to 96-well plates. Then, 10μL of CCK-8 detection reagent (Biosharp #BL1055D) were added at 0, 24, 48, 72, and 96 h and incubated for 2 h. Absorbance values at 450 nm were then detected.

For clone detection, BMSCs (400 cells/well) were inoculated into a 6-well plate and incubated for 2 weeks. Then, 4% PFA was used for fixation for 10 min, and crystal violet staining was performed.

For the EdU test, cells were allowed to grow to a 60% confluence before adding the EdU staining solution (Biosharp #BL916A). Cells were then incubated for 2 h and then digested with trypsin and fixed with a fixing solution for 15 min. After fixation, the penetration solution was added, and the cultures were incubated at room temperature for 15 min. The reaction solution was then added and incubated in a dark room at room temperature for 30 min. After incubation, flow cytometry was performed.

### piRNAs mimic and inhibitors transfection

A solution (200 nM) of mimics and inhibitors was prepared by combining 5 μL of mimics or inhibitors with 500 μL of opti-MEM (Gibco #31,985–070). To make the transfection solution, 5 μL of transfection reagent (Biosharp #BL623A) was added to opti-MEM and allowed to stand for 5 min at room temperature. The transfection reagent was then added to oligonucleotide-containing opti-MEM, and the resulting solutions were allowed to stand for 25 min at room temperature. The solution was then inoculated onto the culture plate and incubated in the incubator for 4 h, after which, the cell culture medium was replaced.

### Immunofluorescence analysis

Cells were fixed with 4% PFA, washed with TBST twice, and sealed by adding 5% BAS for 1 h. The cells were then incubated with the Collagen Ⅰ primary antibody (Abcam #ab138492) solution at 4 ℃ overnight. Afterward, the cells were washed twice with TBST and incubated with the secondary antibody (Abcam #ab288151) solution at room temperature for 1 h. After incubation, the cells were washed twice with TBST, incubated with the DAPI staining solution (Biosharp #BL105B) for 10 min, washed twice with TBST, and sealed with an anti-quenching sealing agent.

### Osteogenic differentiation assay

For ALP staining, 4% PFA was used to fix the cells, which was removed before adding an appropriate amount of BCIP/NBT staining working solution (Beyotime # C3206). The cells were then incubated in the dark at room temperature for 30 min.

For the quantitative detection of ALP activity, cells were collected via centrifugation, and the supernatant was discarded. After repeated freezing and thawing, cells were centrifuged at 4 ℃ and 12,000 rpm for 10 min. The ALP reaction solution (Biosharp #BL862B) was added to the supernatant, and the solution was incubated at 37 ℃ for 20 min. The absorbance value was then obtained at 405 nm, which was used to calculate the relative activity of ALP.

For alizarin red staining and analysis, after cell fixation, the alizarin red staining solution (Biosharp #BL1118A) was added to the cells at room temperature for 10 min, then washed twice with phosphate-buffered saline, photographed, and observed, and 2 mL 100 mM sodium hydroxide solution was added 10 times, and the absorbance value was measured at 584 nm.

### Quantitative PCR analysis

Total RNA was extracted from the BMSCs using the TRIzol reagent. The EZ-press microRNA reverse transcription kit (EZBioscience # EZB-miRT2-plus-L) was used to reverse transcribe the piRNA into cDNA, and the HiScript III 1st Strand cDNA Synthesis Kit (Vazyme # R312-01) was used to reverse transcribe the mRNA into cDNA. The Taq Pro Universal SYBR qPCR Master Mix (# Q712; Vazyme) kit was used for qPCR that was run using a LightCycler480 thermocycler with the following conditions: 95 ℃, 30 s, 1 cycle; 95 ℃, 10 s, 60 ℃, 30 s, 60 cycles; and 95 ℃, 15 s, 60 ℃, 60 s, 95 ℃, 15 s, 1 cycle. Using U6 and GAPDH as internal parameters, the relative expression was calculated according to the 2^−△△Ct^ method. The qPCR primers are listed in Supplementary Table S1.

### Bone mass analysis

This study was approved by the Animal Ethics Committee of Xinhua Hospital, which is affiliated with the Shanghai Jiaotong University School of Medicine. A glucocorticoid-induced osteoporosis model was established using 8-week-old C57BL/6 J male mice by subcutaneously injecting dexamethasone sodium phosphate (50 mg/kg body weight) for 4 weeks. The 12-week-old glucocorticoid-induced osteoporosis mice were then randomly divided into three groups (six mice per group).. The tibia and femur of the NC group were each injected with 25 μL phosphate-buffered saline, while those of the mimic NC group were injected with 1 × 10^5^ BMSCs, and those of the DQ690018 mimic group were injected with 1 × 10^5^ DQ690018-modified BMSCs. One month after the injection, the mice were euthanized, and the tibia and femur were removed for micro-CT detection (Sanying #nanoVoxel-1000). After detection, the tibia and femur were decalcified and embedded in paraffin, then 10-μm paraffin section cuts were created and hematoxylin–eosin staining was performed.

### Statistic analysis

The values were presented in the form of means ± S.D., and the statistical difference between the two groups was compared using the student’s t-test. The following p-values ( listed in Supplementary Table S2) were considered statistically significant: "*" indicates that p < 0.05, and "**" indicates that p < 0.01.

## Results

### piRNA DQ690018 was upregulated during BMSC proliferation

**Fig. 1:**
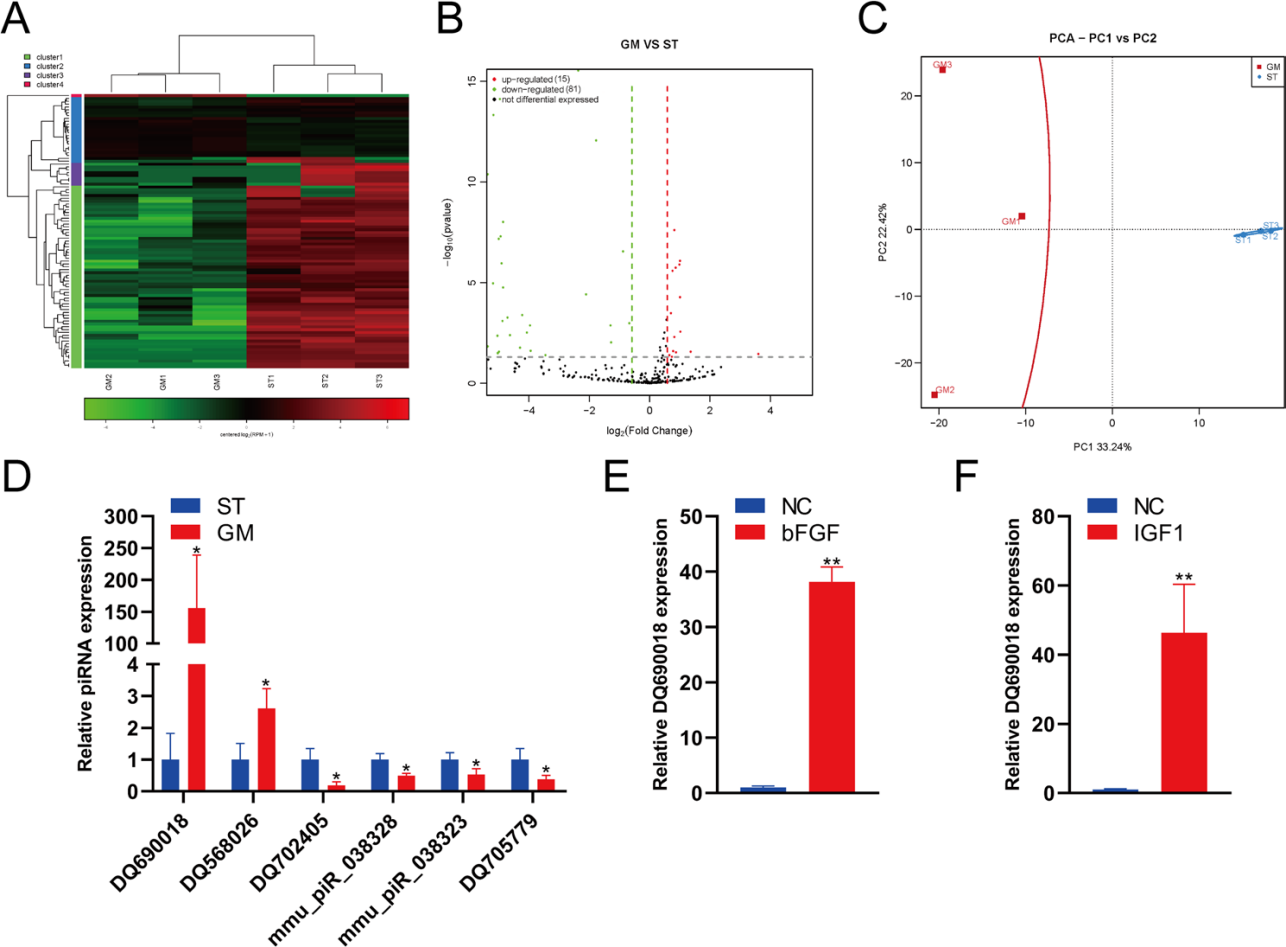
PiRNA DQ690018 upregulated during the proliferation of BMSCs. **A** Heatmap showed the expression of piRNA in BMSCs in ST and GM group, **B** Volcano map showed the expression of piRNA in BMSCs in ST and GM group, **C** PCA analysis the expression of PCA in ST and GM group. **D** The expression of piRNA in ST and GM group were detected by qPCR, **E** The expression of DQ690018 in BMSCs stimulated by bFGF were detected by qPCR, **F**The expression of DQ690018 in BMSCs stimulated by bFGF were detected by qPCR. All data were obtained from at least three independent experiments (biological replicates, with three technical replicates per experiment). Data are presented as the mean ± standard error. ‘*’indicated p < 0.05, ‘**’indicated p < 0.01

**Fig. 2:**
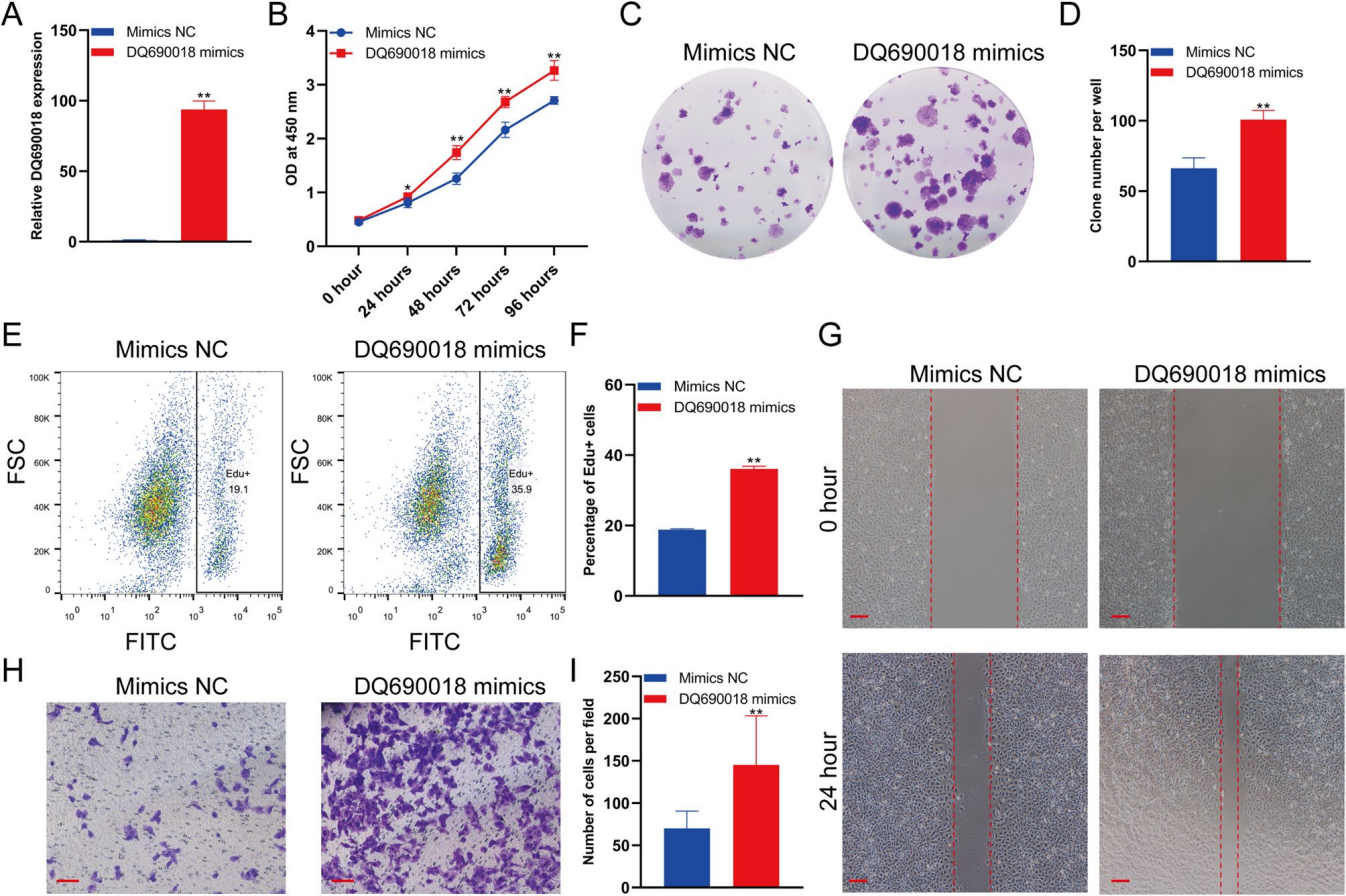
PiRNA DQ690018 overexpression promoted the proliferation and migration of BMSCs. **A** The expression of DQ690018 in BMSCs transfected with DQ690018 mimics detected by qPCR, **B** The proliferation of BMSCs transfected with DQ690018 mimics detected by CCK-8, **C** The proliferation of BMSCs transfected with DQ690018 mimics detected by clone formation, **D** Quantitative analysis of clone formation. **E** The Edu positive cells were detected by flow cytometry, **F** Quantitative analysis of Edu positive cells. **G** The migration of BMSCs were detected by scratch test, scale bar 200 μm, **H** The migration of BMSCs were detected by transwell, scale bar 100 μm, **I** Quantitative analysis of transwell. All data were obtained from at least three independent experiments (biological replicates, with three technical replicates per experiment). Data are presented as the mean ± standard error. ‘*’indicated p < 0.05, ‘**’indicated p < 0.01

Small RNA sequencing was used to analyze piRNA expression during BMSC proliferation. The heatmap and volcano map show significantly different piRNA expression in BMSCs between the GM and ST groups. Compared to ST group BMSCs, 15 piRNAs were upregulated, and 81 piRNAs were downregulated in GM group BMSCs (Fig. 1A and B). Principal component analysis showed that the piRNA expression was significantly different between BMSCs from the GM and ST groups (Fig. 2C). qPCR analysis showed that the expression of DQ690018 and DQ568026 was upregulated and that of DQ702405, mmu_piR_038328, mmu_piR_038323, and DQ705779 was downregulated in the GM group compared with that in ST group BMSCs (Fig. 2D). DQ690018 expression in BMSCs was upregulated by bFGF and IGF1 stimulation (Fig. 2E and F).

### piRNA DQ690018 overexpression promoted the proliferation and migration of BMSCs

DQ690018 mimics were used to overexpress DQ690018 in BMSCs. qPCR analysis showed that DQ690018 expression was upregulated in the BMSCs of the DQ690018 mimic group (Fig. 2A). DQ690018 overexpression promoted the proliferation of BMSCs, as detected through the CCK-8 assay (Fig. 2B). The number and size of BMSC clones in the DQ690018 mimic group were higher than those in the mimic NC group (Fig. 2C and D). The percentage of EdU-positive BMSCs in the DQ690018 mimic group was higher than that in the mimic NC group (Fig. 2E and F). The wound healing assay showed that DQ690018 overexpression promoted BMSC migration (Fig. 2G). The number of migrated cells significantly increased in the DQ690018 mimic group compared to that in the mimic NC group, as detected via the Transwell assay (Fig. 2H and I).

### piRNA DQ690018 knockdown inhibited the proliferation and migration of BMSCs

**Fig. 3:**
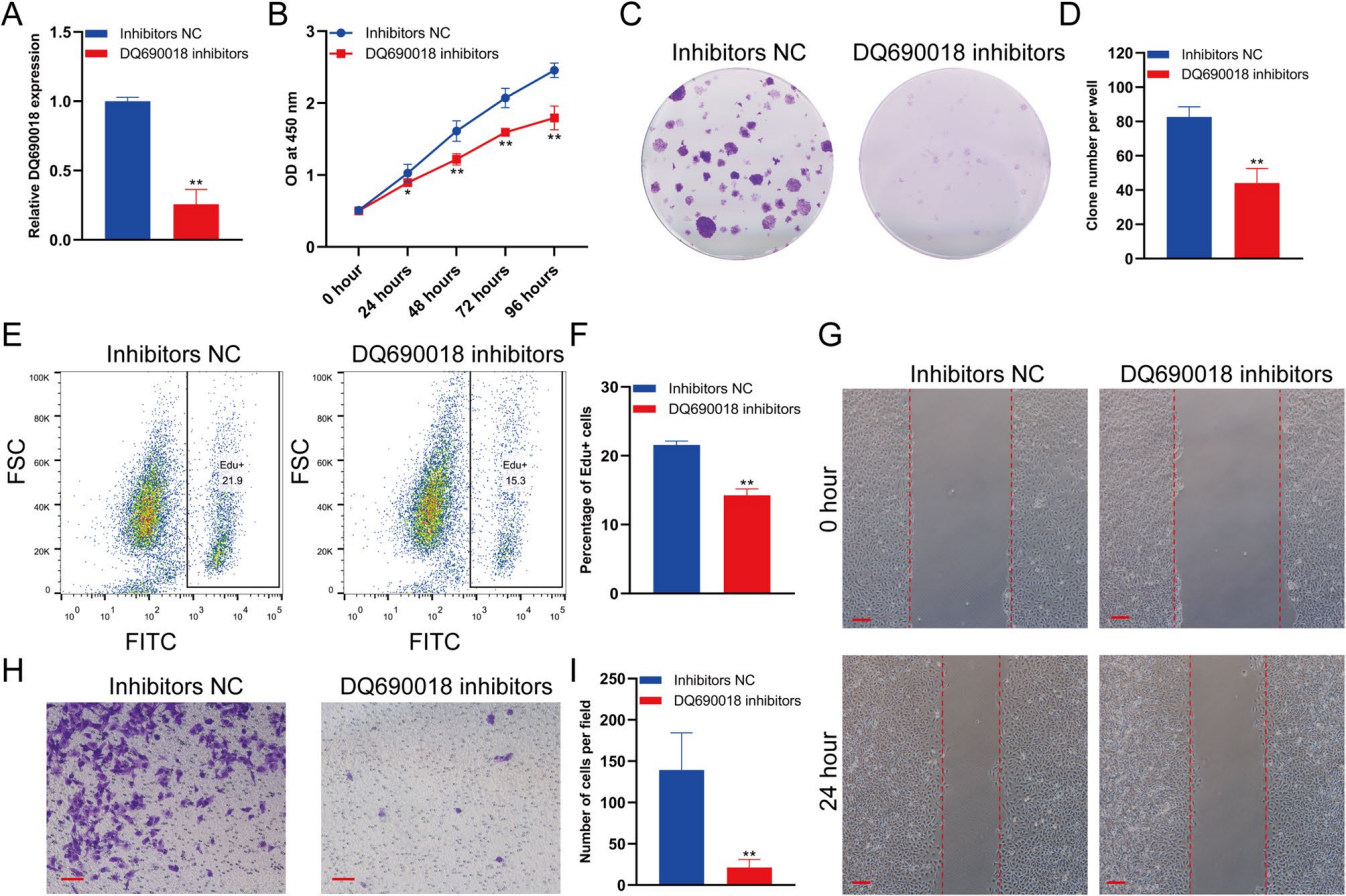
PiRNA DQ690018 knockdown inhibited the proliferation and migration of BMSCs. **A**The expression of DQ690018 in BMSCs transfected with DQ690018 inhibitors detected by qPCR, **B** The proliferation of BMSCs transfected with DQ690018 inhibitors detected by CCK-8, **C** The proliferation of BMSCs transfected with DQ690018 inhibitors detected by clone formation, **D** Quantitative analysis of clone formation. **E** The Edu positive cells were detected by flow cytometry, **F** Quantitative analysis of Edu positive cells. **G** The migration of BMSCs were detected by scratch test, scale bar 200 μm, **H** The migration of BMSCs were detected by transwell, scale bar 100 μm, **I** Quantitative analysis of transwell. All data were obtained from at least three independent experiments (biological replicates, with three technical replicates per experiment). Data are presented as the mean ± standard error. ‘*’indicated p < 0.05, ‘**’indicated p < 0.01

DQ690018 inhibitors were used to knock down DQ690018 in BMSCs. The qPCR analysis revealed that DQ690018 expression was downregulated in the DQ690018 inhibitor group (Fig. 3A). Furthermore, DQ690018 knockdown inhibited the proliferation of BMSCs, as detected via the CCK-8 assay (Fig. 3B). The number and size of the BMSC clones in the DQ690018 inhibitor group were lower than those in the inhibitor NC group (Fig. 3C and D). The percentage of EdU-positive BMSCs in the DQ690018 inhibitor group was lower than in the NC inhibitor group (Fig. 3E and F). The wound healing assay showed that DQ690018 knockdown inhibited the migration of BMSCs (Fig. 3G). The number of migrated cells decreased significantly in the DQ690018 inhibitor group compared to that in the NC inhibitor group, as detected via the Transwell assay (Fig. 3H and I).

### piRNA DQ690018 regulated the expression of p21 and Vcl

**Fig. 4:**
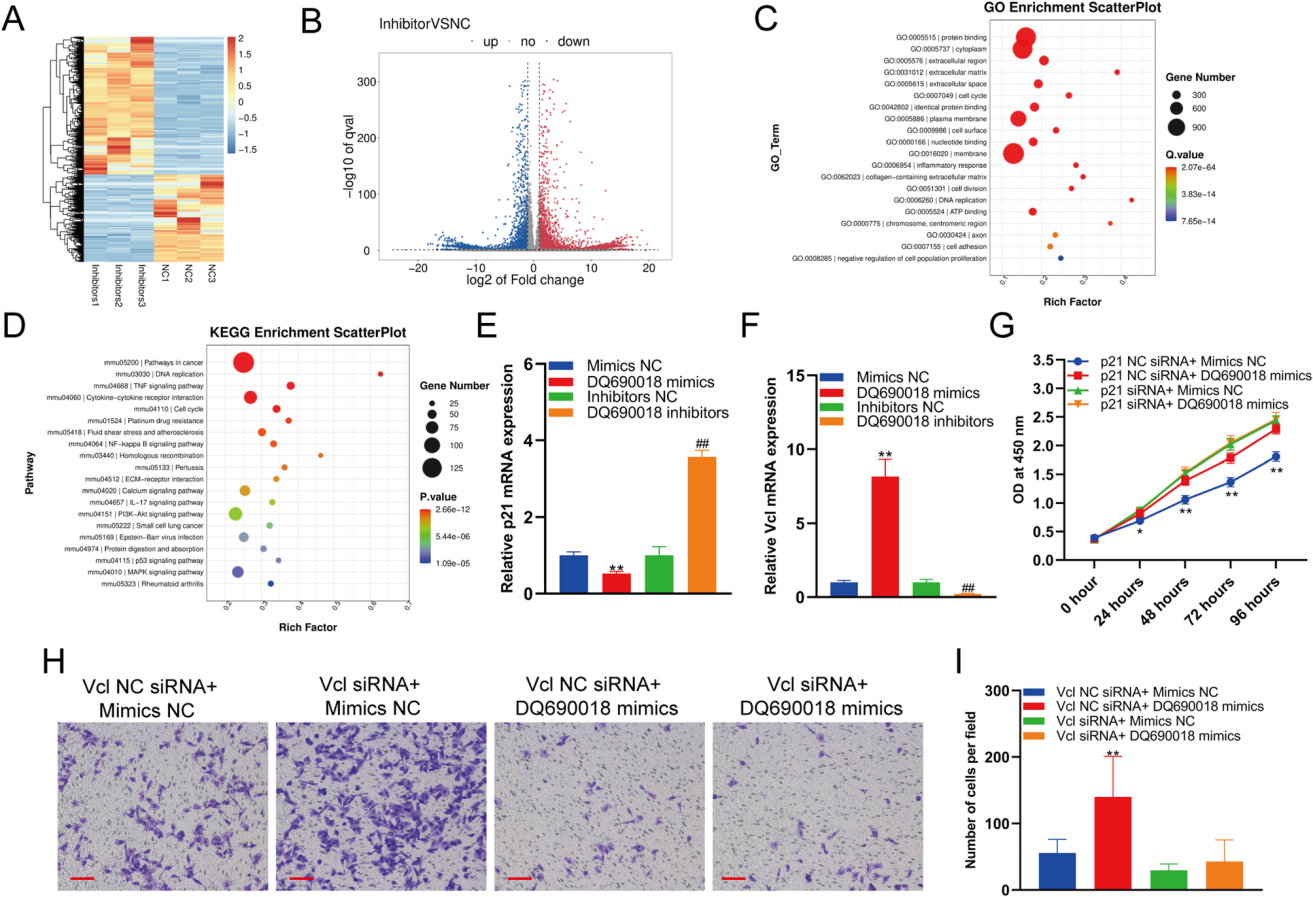
PiRNA DQ690018 regulated the expression of p21 and Vcl. **A**Heatmap showed the different expressed genes in BMSCs with DQ690018 knockdown, **B** Volcano map showed the different expressed genes in BMSCs with DQ690018 knockdown, **C** GO enrichment analysis of different expressed genes in BMSCs with DQ690018 knockdown, **D** KEGG enrichment analysis of different expressed genes in BMSCs with DQ690018 knockdown, **E** The expression of p21 mRNA in BMSCs with DQ690018 overexpression or knockdown were detected with qPCR, **F** The expression of Vcl mRNA in BMSCs with DQ690018 overexpression or knockdown were detected with qPCR, **G**The proliferation of BMSCs with DQ690018 overexpression under p21 knockdown were detected with CCK-8, **E** The migration of BMSCs with DQ690018 overexpression under Vcl knockdown were detected with transwell, scale bar 100 μm, **F** Quantitative analysis of transwell. All data were obtained from at least three independent experiments (biological replicates, with three technical replicates per experiment). Data are presented as the mean ± standard error. ‘*’indicated p < 0.05, ‘**’indicated p < 0.01

To identify the target of DQ690018, DQ690018 was knocked down in BMSCs, and mRNA sequencing was used to detect changes in mRNA expression in BMSCs. The heatmap and volcano map show significant differential mRNA expression in BMSCs between the DQ690018 inhibitor group and the inhibitor NC group (Fig. 4A and B). GO enrichment analysis showed that the differentially expressed genes were significantly enriched in the cell cycle, cell division, DNA replication, extracellular matrix, and collagen-containing extracellular matrix, which are related to cell proliferation, migration, and osteogenic differentiation (Fig. 4C). KEGG enrichment analysis revealed that the significant differentially expressed genes were enriched in pathways involved in cancer, the cell cycle, ECM-receptor interaction, DNA replication, and rheumatoid arthritis, which are related to cell proliferation, migration, and osteogenic differentiation (Fig. 4D). qPCR analysis showed that DQ690018 overexpression inhibited the expression of p21 and DQ690018 knockdown promoted p21 expression (Fig. 4E). In contrast, DQ690018 overexpression and knockdown promoted and inhibited Vcl expression, respectively (Fig. 4F). To detect the regulatory effect of DQ690018 on BMSC proliferation, p21 expression in BMSCs was knocked down using a p21-specific siRNA. CCK-8 analysis showed that the promoting effect of DQ690018 on BMSC proliferation was polished when the p21 was knocked down (Fig. 4G). CCK-8 analysis showed that DQ690018 promoted the migration of BMSCs when Vcl expression was knocked down (Fig. 4H and I).

### piRNA DQ690018 regulated the osteogenic differentiation of BMSCs

**Fig. 5:**
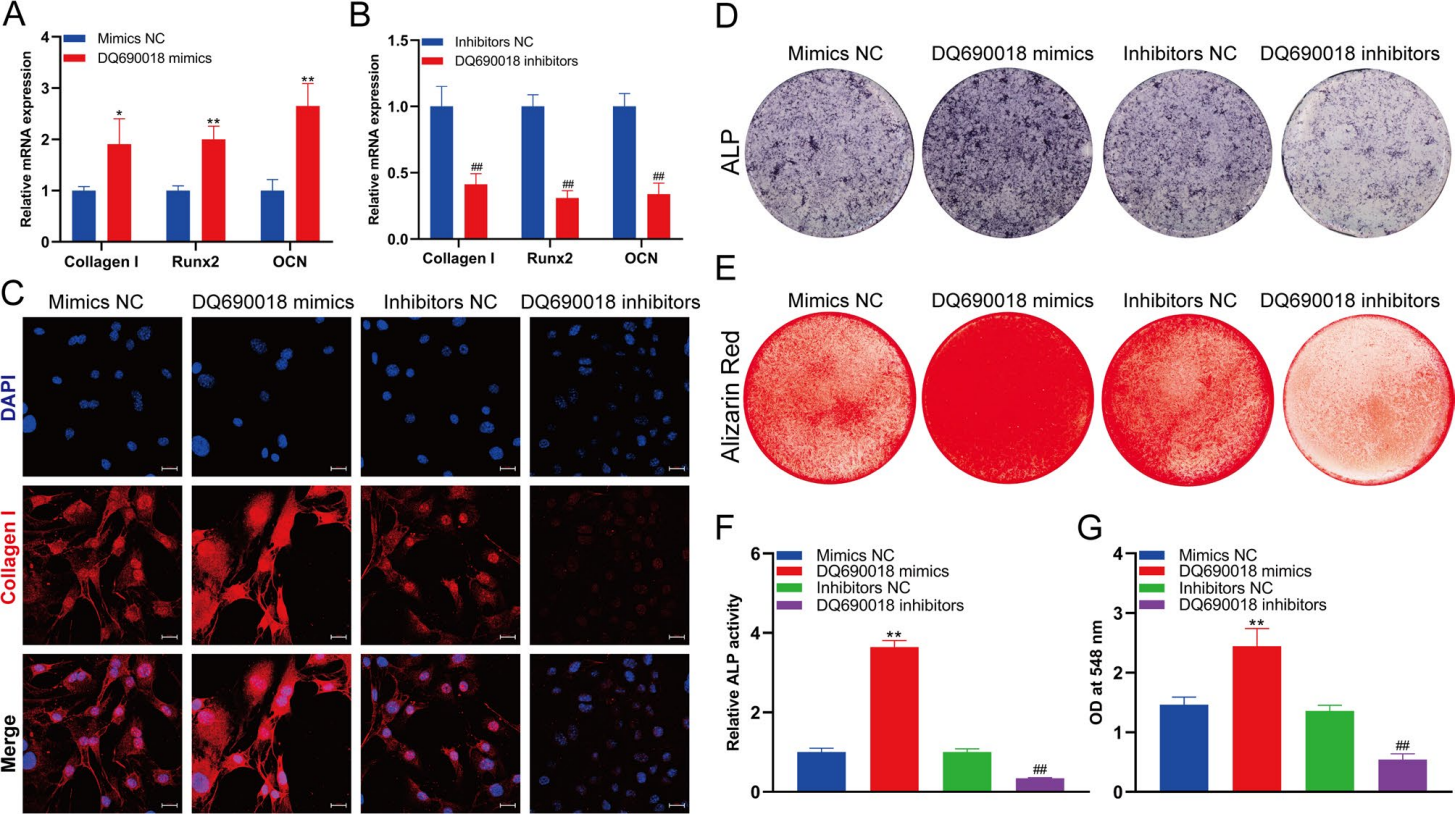
PiRNA DQ690018 regulated the osteogenic differentiation of BMSCs. **A**The Collagen I, Runx2 and OCN mRNA expression in BMSCs with DQ690018 knockdown were detected with qPCR, **B** The Collagen I, Runx2 and OCN mRNA expression in BMSCs with DQ690018 overexpression were detected with qPCR, **C** Immunofluorescence analysis of the Collagen I mRNA expression in BMSCs with DQ690018 knockdown or overexpression, scale bar 10 μm, **D** ALP staining analysis of osteogenic differentiation of BMSCs with DQ690018 knockdown or overexpression, **E** Alizarin Red staining analysis of osteogenic differentiation of BMSCs with DQ690018 knockdown or overexpression, **F** Quantitative analysis of ALP activity, **G** Quantitative analysis of Alizarin red. All data were obtained from at least three independent experiments (biological replicates, with three technical replicates per experiment). Data are presented as the mean ± standard error. ‘*’indicated p < 0.05, ‘**’indicated p < 0.01 DQ690018 mimics group VS Mimimcs NC group. ‘##’indicated p < 0.01 DQ690018 inhibitors group VS Inhibitors NC group

The mRNA sequencing results showed that DQ690018 also regulated the expression of Col1al, ALP, IBSP, and IHH, which are associated with the osteogenic differentiation of BMSCs. The qPCR analysis showed that DQ690018 overexpression promoted the mRNA expression of collagen I, Runx2, and OCN (Fig. 5A) while knockdown inhibited their expression (Fig. 5B). Immunofluorescence analysis showed that DQ690018 overexpression and knockdown promoted and inhibited Collagen Ⅰ expression, respectively, in BMSCs (Fig. 5C). Similarly, DQ690018 overexpression increased ALP activity while its knockdown decreased ALP activity in BMSCs (Fig. 5D and F). Alizarin red staining results showed that DQ690018 overexpression and knockdown, respectively, increased and decreased the extracellular matrix calcium nodules in BMSCs (Fig. 5E and G).

### BMSCs with DQ690018 overexpression increased the bone mass

**Fig. 6:**
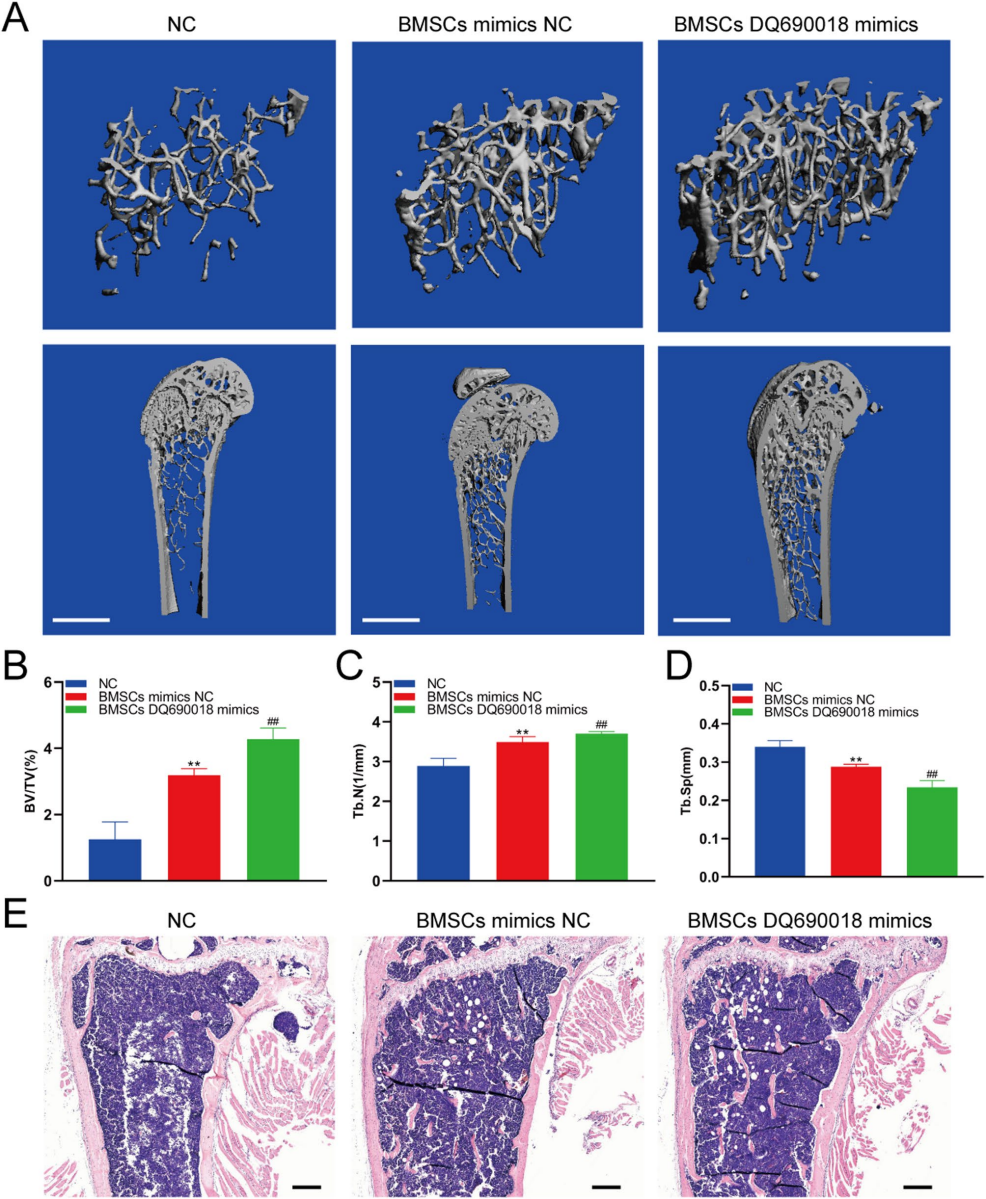
BMSCs with DQ690018 overexpression increased the bone mass. **A**The microCT detection of bone mass with BMSCs treatment, scale bar 1 mm, **B** Quantitative analysis of BV/TV, **C** Quantitative analysis of Tb.N, **D** Quantitative analysis of Tb.Sp, **E** H&E staining analysis the bone mass, scale bar 200 μm. All data were obtained from six mice. Data are presented as the mean ± standard error. ‘**’indicated p < 0.01 BMSCs mimics NC group VS NC group. ‘##’indicated p < 0.01 BMSCs DQ690018 mimics group VS BMSCs mimis NC group

BMSC-based cell therapy is an important treatment for osteoporosis. BMSCs transfected with NC and DQ690018 mimics were injected into the bone marrow cavity of mice. Micro-CT analysis showed that BMSCs transfected with the DQ690018 mimic had a stronger ability to increase bone mass (Fig. 6A). Compared to the NC group, BV/TV and Tb.N values were higher, while Tb.Sp was lower in the DQ690018 group (Fig. 6B–D). H&E staining also showed that the bone mass in the DQ690018 group was higher than that in the Mimic NC group (Fig. 6E).

## Discussion

Bone marrow mesenchymal stem cell transplantation is one of the most promising treatments for nonunion fractures and osteoporosis[22]. The number of active cells in the target tissue is a key factor in achieving good results in the treatment of degenerative or aplastic diseases by BMSC transplantation[23]. The microenvironment at the implantation site also significantly impacts BMSC function[24]. For instance, the high glucose microenvironment in patients with diabetes inhibits the proliferation and migration of BMSCs, resulting in decreased bone regeneration ability [25]. Molecules expressed on the surface of BMSCs, such as SDF-1, CXCR4, and CCR1, contribute to their migration and homing. Therefore, increasing the expression of these molecules on the surface of BMSCs may enhance the efficacy of BMSC therapy. A high SFD1 concentration enhanced the proliferation, migration, and osteogenic differentiation of mouse BMSCs by activating the Wnt/β-catenin signaling pathway [26]. HIF-1α prolyl hydroxylase inhibitor promotes fracture healing by enhancing the proliferation and migration of BMSCs [27]. The bFGF is a basic member of the fibroblast growth factor family that enhances the proliferation and migration of BMSCs [28–30]. IGF-1 plays an important role in regulating cell proliferation, differentiation, and migration and can enhance the proliferation and migration abilities of the BMSCs [31, 32]. However, some current strategies for enhancing BMSC function have inherent limitations [33, 34]. These methods cannot accurately optimize the number of cytokines released, resulting in certain side effects. Genetic modifications cannot prevent the side effects caused by potential gene mutations [35].

In this study, we found that DQ690018 promoted the proliferation, migration, and osteogenic differentiation of BMSCs. DQ690018 is an important new target molecule that can enhance the function of BMSCs. A study on an osteoporosis treatment that utilized transplanted BMSCs modified by DQ690018 mimics found that the modified BMSCs have a stronger osteogenic ability; this provided a new optimization condition for BMSC-based cell therapy.

piRNAs play an important role in maintaining genomic stability, silencing the expression of non-self sequences and maintaining male reproduction [36]. Initially, piRNAs were thought to be expressed only in male germ cells [37]. Further studies have shown that piRNAs are also expressed in adult, stem, and tumor cells. There are relatively few studies on piRNA regulation of BMSC function. piR-63049 function is significantly increased in BMSCs derived from OVX rats, and the inhibition of the Wnt2b/β-catenin signaling pathway by piR-63049 also inhibits BMSC osteogenic differentiation [38]. PiRNA-36741 promotes osteogenic BMSC differentiation by inhibiting METTL3-mediated m6A methylation of BMP2 mRNA [39]. The expression of piRNAs in human BMSCs also changes significantly while aging [40]. Furthermore, DQ592931 and DQ571813 expression is significantly decreased during the chondrogenic differentiation of human BMSCs [41]. In this study, we found that the expression of DQ690018 increased during BMSC proliferation. BFGF and IGF-1 significantly increased the expression of DQ690018 in BMSCs. The regulatory effects of bFGF and IGF-1 on the function of BMSCs may be partially dependent on the increased DQ690018 expression. The effect of BMSC-based cell therapy on bone damage repair partly depends on cytokines and exosomes released from human BMSCs. It has been found that exosomes from BMSCs contain piRNA [20, 42]. In this study, we found that DQ690018 mimic-modified BMSCs significantly increased the bone mass of mice. Whether the exosomes from BMSCs contained DQ690018, as well as the effect of DQ690018 on other bone regeneration-related cells, need to be further investigated.

Through mRNA sequencing and cell function detection, we found that DQ690018 regulated the expression of p21 and Vcl in BMSCs. p21 is one of the most important downstream genes of p53. p21 could combine with almost every type of cyclin-CDK complex and widely inhibited all types of cyclin-CDK complexes, thereby stopping the cell cycle at the G1 phase and inhibiting DNA replication. Inhibiting p21 expression could promote the proliferation and osteogenic differentiation of BMSCs and maintain their characteristics [43]. Mechanical signals promote the osteogenic differentiation of BMSCs by inhibiting p21 expression [44]. BMP9 alleviates osteoblast aging and promotes osteoblastic differentiation by inhibiting p21 [45]. Thus, the DQ690018/p21 signaling pathway may be a new target for regulating the proliferation and osteogenic differentiation of BMSCs. Vinculin, also known as an adhesion spot protein, is a cytoskeletal protein that is concentrated in cell–cell and cell–matrix adhesion sites and participates in cell adhesion, extension, migration, cell morphology maintenance, mechanochemical signal transduction, and other physiological processes. Previous studies have shown that vinculin is abnormally expressed in highly invasive and metastatic tumor cells and participates in other signaling pathways that affect the occurrence and development of malignant tumors [46, 47]. We found that DQ690018 significantly promoted the expression of vinculin in BMSCs, and DQ690018 could promote their migration by regulating vinculin expression.

While our study reveals novel insights into the role of piRNA DQ690018 in regulating BMSC proliferation, migration, and osteogenic differentiation, several limitations must be acknowledged. First, our experiments primarily focused on short-term endpoints, leaving the long-term effects of DQ690018 modulation on BMSC behavior and bone regeneration unaddressed. Second, although the use of piRNA mimics and inhibitors enabled us to elucidate the functional roles of DQ690018, these approaches may carry inherent off-target effects that could influence the observed cellular responses. Furthermore, while our in vitro assays and acute in vivo interventions provide valuable mechanistic insights, they may not fully replicate the complex in vivo microenvironment of bone marrow. Future studies employing more targeted gene-editing techniques, extended follow-up periods, and larger cohorts are necessary to validate our findings and clarify the clinical relevance of DQ690018 in bone regenerative therapies.

## Conclusion

DQ690018 overexpression significantly improved the proliferation, migration, and osteogenic differentiation of BMSCs. Thus, DQ690018 mimics that modify BMSCs can be used as a new optimization strategy for BMSC-based cell therapy.

## Supplementary Information

Below is the link to the electronic supplementary material.

Supplementary Material 1

Supplementary Material 2

## Data Availability

The data that support the findings of this study are available from the corresponding author upon reasonable request.
